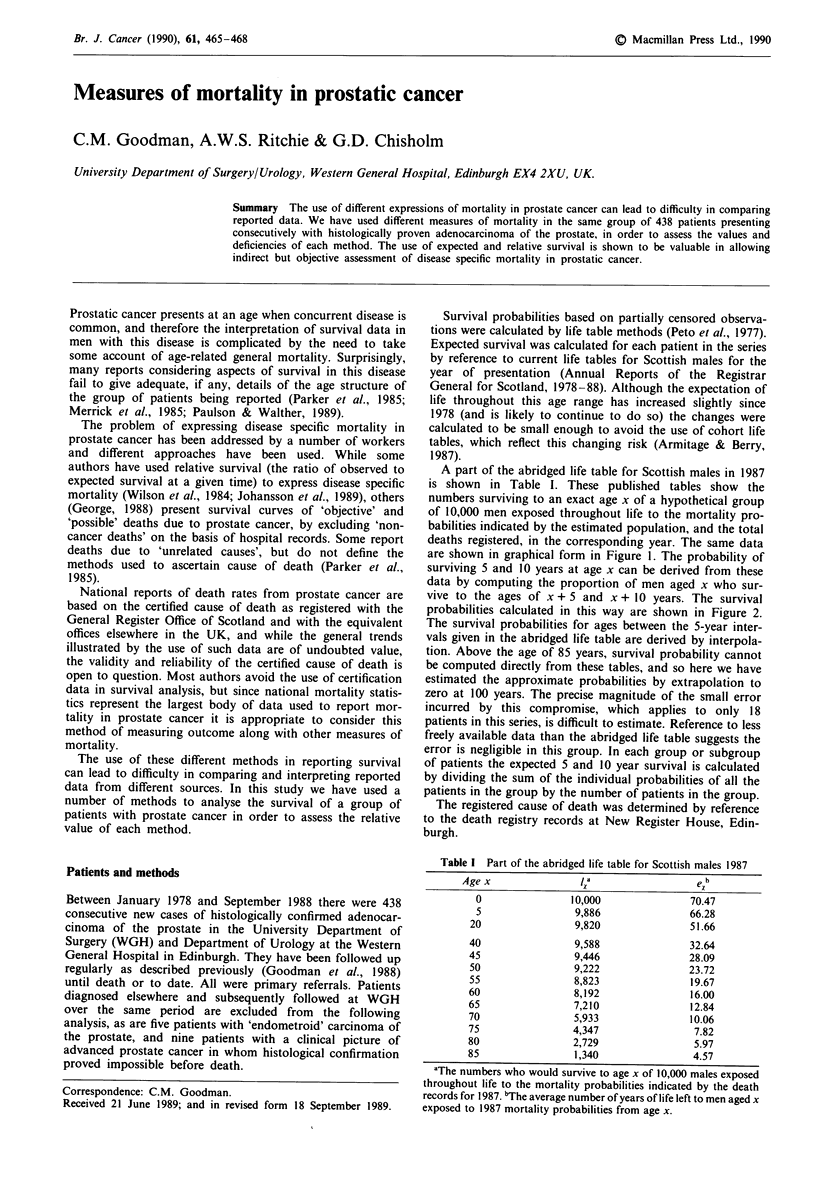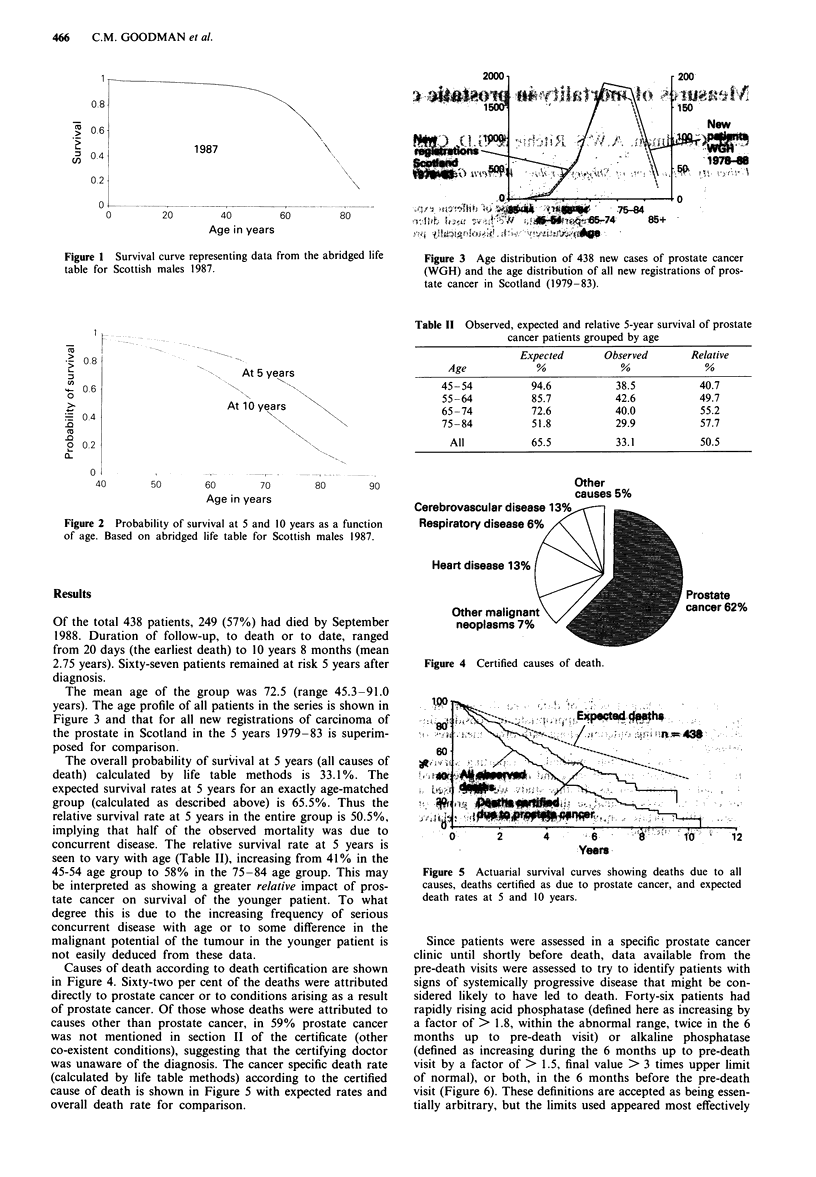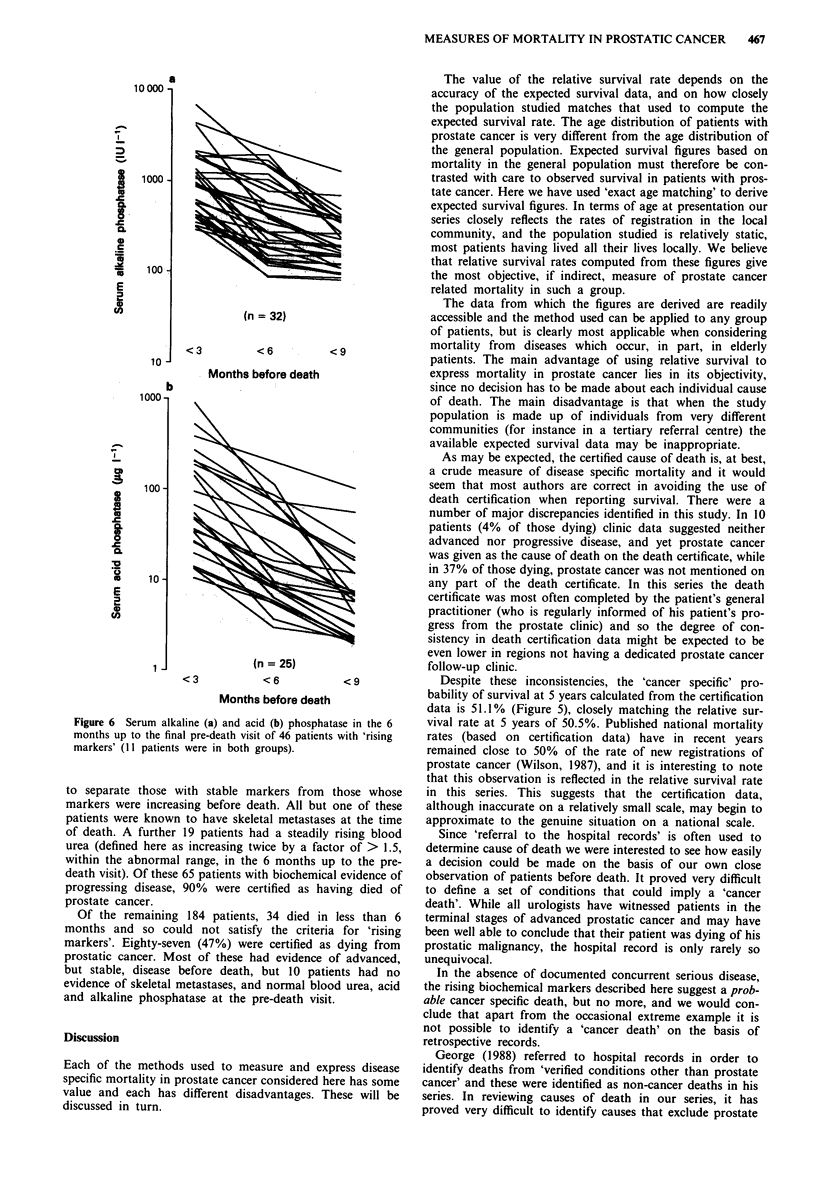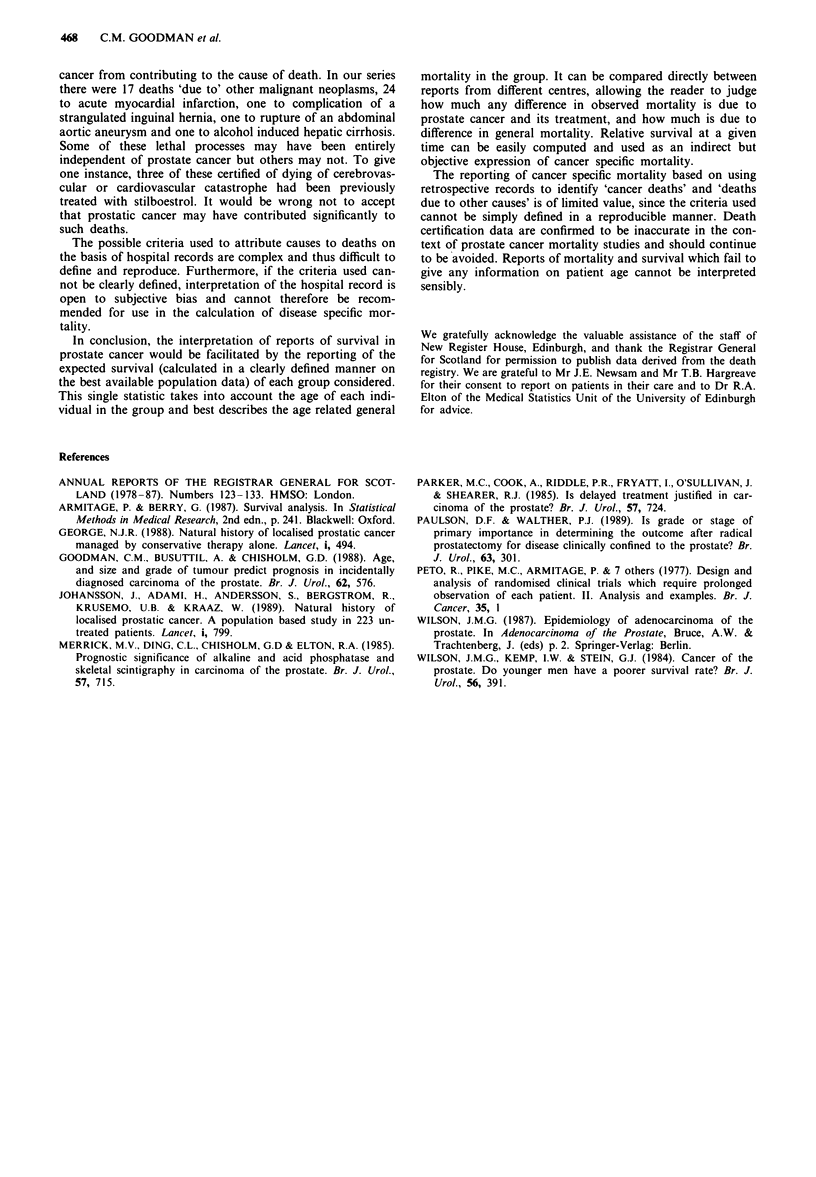# Measures of mortality in prostatic cancer.

**DOI:** 10.1038/bjc.1990.102

**Published:** 1990-03

**Authors:** C. M. Goodman, A. W. Ritchie, G. D. Chisholm

**Affiliations:** University Department of Surgery/Urology, Western General Hospital, Edinburgh, UK.

## Abstract

The use of different expressions of mortality in prostate cancer can lead to difficulty in comparing reported data. We have used different measures of mortality in the same group of 438 patients presenting consecutively with histologically proven adenocarcinoma of the prostate, in order to assess the values and deficiencies of each method. The use of expected and relative survival is shown to be valuable in allowing indirect but objective assessment of disease specific mortality in prostatic cancer.


*Br. J. Cancer* (1990), **61**, 465–468 © Macmillan Press Ltd., 1990

### Measures of mortality in prostatic cancer

C.M. Goodman, A.W.S. Ritchie & G.D. Chisholm

*University Department of Surgery/Urology, Western General Hospital, Edinburgh EX4 2XU, UK.*

**Summary** The use of different expressions of mortality in prostate cancer can lead to difficulty in comparing reported data. We have used different measures of mortality in the same group of 438 patients presenting consecutively with histologically proven adenocarcinoma of the prostate, in order to assess the values and deficiencies of each method. The use of expected and relative survival is shown to be valuable in allowing indirect but objective assessment of disease specific mortality in prostatic cancer.

Prostatic cancer presents at an age when concurrent disease is common, and therefore the interpretation of survival data in men with this disease is complicated by the need to take some account of age-related general mortality. Surprisingly, many reports considering aspects of survival in this disease fail to give adequate, if any, details of the age structure of the group of patients being reported (Parker *et al.*, 1985; Merrick *et al.*, 1985; Paulson & Walther, 1989).

The problem of expressing disease specific mortality in prostate cancer has been addressed by a number of workers and different approaches have been used. While some authors have used relative survival (the ratio of observed to expected survival at a given time) to express disease specific mortality (Wilson *et al.*, 1984; Johansson *et al.*, 1989), others (George, 1988) present survival curves of 'objective' and 'possible' deaths due to prostate cancer, by excluding 'non-cancer deaths' on the basis of hospital records. Some report deaths due to 'unrelated causes', but do not define the methods used to ascertain cause of death (Parker *et al.*, 1985).

National reports of death rates from prostate cancer are based on the certified cause of death as registered with the General Register Office of Scotland and with the equivalent offices elsewhere in the UK, and while the general trends illustrated by the use of such data are of undoubted value, the validity and reliability of the certified cause of death is open to question. Most authors avoid the use of certification data in survival analysis, but since national mortality statistics represent the largest body of data used to report mortality in prostate cancer it is appropriate to consider this method of measuring outcome along with other measures of mortality.

The use of these different methods in reporting survival can lead to difficulty in comparing and interpreting reported data from different sources. In this study we have used a number of methods to analyse the survival of a group of patients with prostate cancer in order to assess the relative value of each method.

### Patients and methods

Between January 1978 and September 1988 there were 438 consecutive new cases of histologically confirmed adenocarcinoma of the prostate in the University Department of Surgery (WGH) and Department of Urology at the Western General Hospital in Edinburgh. They have been followed up regularly as described previously (Goodman *et al.*, 1988) until death or to date. All were primary referrals. Patients diagnosed elsewhere and subsequently followed at WGH over the same period are excluded from the following analysis, as are five patients with 'endometroid' carcinoma of the prostate, and nine patients with a clinical picture of advanced prostate cancer in whom histological confirmation proved impossible before death.

Correspondence: C.M. Goodman.
Received 21 June 1989; and in revised form 18 September 1989.

Survival probabilities based on partially censored observations were calculated by life table methods (Peto *et al.*, 1977). Expected survival was calculated for each patient in the series by reference to current life tables for Scottish males for the year of presentation (Annual Reports of the Registrar General for Scotland, 1978–88). Although the expectation of life throughout this age range has increased slightly since 1978 (and is likely to continue to do so) the changes were calculated to be small enough to avoid the use of cohort life tables, which reflect this changing risk (Armitage & Berry, 1987).

A part of the abridged life table for Scottish males in 1987 is shown in Table I. These published tables show the numbers surviving to an exact age $x$ of a hypothetical group of 10,000 men exposed throughout life to the mortality probabilities indicated by the estimated population, and the total deaths registered, in the corresponding year. The same data are shown in graphical form in Figure 1. The probability of surviving 5 and 10 years at age $x$ can be derived from these data by computing the proportion of men aged $x$ who survive to the ages of $x + 5$ and $x + 10$ years. The survival probabilities calculated in this way are shown in Figure 2. The survival probabilities for ages between the 5-year intervals given in the abridged life table are derived by interpolation. Above the age of 85 years, survival probability cannot be computed directly from these tables, and so here we have estimated the approximate probabilities by extrapolation to zero at 100 years. The precise magnitude of the small error incurred by this compromise, which applies to only 18 patients in this series, is difficult to estimate. Reference to less freely available data than the abridged life table suggests the error is negligible in this group. In each group or subgroup of patients the expected 5 and 10 year survival is calculated by dividing the sum of the individual probabilities of all the patients in the group by the number of patients in the group.

The registered cause of death was determined by reference to the death registry records at New Register House, Edinburgh.

**Table I** Part of the abridged life table for Scottish males 1987

| *Age x* | $l_x$[a] | $e_x$[b] |
|---|---|---|
| 0 | 10,000 | 70.47 |
| 5 | 9,886 | 66.28 |
| 20 | 9,820 | 51.66 |
| 40 | 9,588 | 32.64 |
| 45 | 9,446 | 28.09 |
| 50 | 9,222 | 23.72 |
| 55 | 8,823 | 19.67 |
| 60 | 8,192 | 16.00 |
| 65 | 7,210 | 12.84 |
| 70 | 5,933 | 10.06 |
| 75 | 4,347 | 7.82 |
| 80 | 2,729 | 5.97 |
| 85 | 1,340 | 4.57 |

[a]The numbers who would survive to age $x$ of 10,000 males exposed throughout life to the mortality probabilities indicated by the death records for 1987. [b]The average number of years of life left to men aged $x$ exposed to 1987 mortality probabilities from age $x$.

**Figure 1** Survival curve representing data from the abridged life table for Scottish males 1987.

**Figure 2** Probability of survival at 5 and 10 years as a function of age. Based on abridged life table for Scottish males 1987.

### Results

Of the total 438 patients, 249 (57%) had died by September 1988. Duration of follow-up, to death or to date, ranged from 20 days (the earliest death) to 10 years 8 months (mean 2.75 years). Sixty-seven patients remained at risk 5 years after diagnosis.

The mean age of the group was 72.5 (range 45.3–91.0 years). The age profile of all patients in the series is shown in Figure 3 and that for all new registrations of carcinoma of the prostate in Scotland in the 5 years 1979–83 is superimposed for comparison.

The overall probability of survival at 5 years (all causes of death) calculated by life table methods is 33.1%. The expected survival rates at 5 years for an exactly age-matched group (calculated as described above) is 65.5%. Thus the relative survival rate at 5 years in the entire group is 50.5%, implying that half of the observed mortality was due to concurrent disease. The relative survival rate at 5 years is seen to vary with age (Table II), increasing from 41% in the 45-54 age group to 58% in the 75–84 age group. This may be interpreted as showing a greater *relative* impact of prostate cancer on survival of the younger patient. To what degree this is due to the increasing frequency of serious concurrent disease with age or to some difference in the malignant potential of the tumour in the younger patient is not easily deduced from these data.

Causes of death according to death certification are shown in Figure 4. Sixty-two per cent of the deaths were attributed directly to prostate cancer or to conditions arising as a result of prostate cancer. Of those whose deaths were attributed to causes other than prostate cancer, in 59% prostate cancer was not mentioned in section II of the certificate (other co-existent conditions), suggesting that the certifying doctor was unaware of the diagnosis. The cancer specific death rate (calculated by life table methods) according to the certified cause of death is shown in Figure 5 with expected rates and overall death rate for comparison.

**Figure 3** Age distribution of 438 new cases of prostate cancer (WGH) and the age distribution of all new registrations of prostate cancer in Scotland (1979–83).

**Table II** Observed, expected and relative 5-year survival of prostate cancer patients grouped by age

| *Age* | *Expected* % | *Observed* % | *Relative* % |
|---|---|---|---|
| 45–54 | 94.6 | 38.5 | 40.7 |
| 55–64 | 85.7 | 42.6 | 49.7 |
| 65–74 | 72.6 | 40.0 | 55.2 |
| 75–84 | 51.8 | 29.9 | 57.7 |
| All | 65.5 | 33.1 | 50.5 |

**Figure 4** Certified causes of death.

**Figure 5** Actuarial survival curves showing deaths due to all causes, deaths certified as due to prostate cancer, and expected death rates at 5 and 10 years.

Since patients were assessed in a specific prostate cancer clinic until shortly before death, data available from the pre-death visits were assessed to try to identify patients with signs of systemically progressive disease that might be considered likely to have led to death. Forty-six patients had rapidly rising acid phosphatase (defined here as increasing by a factor of > 1.8, within the abnormal range, twice in the 6 months up to pre-death visit) or alkaline phosphatase (defined as increasing during the 6 months up to pre-death visit by a factor of > 1.5, final value > 3 times upper limit of normal), or both, in the 6 months before the pre-death visit (Figure 6). These definitions are accepted as being essentially arbitrary, but the limits used appeared most effectively

**Figure 6** Serum alkaline (**a**) and acid (**b**) phosphatase in the 6 months up to the final pre-death visit of 46 patients with 'rising markers' (11 patients were in both groups).

to separate those with stable markers from those whose markers were increasing before death. All but one of these patients were known to have skeletal metastases at the time of death. A further 19 patients had a steadily rising blood urea (defined here as increasing twice by a factor of $> 1.5$, within the abnormal range, in the 6 months up to the pre-death visit). Of these 65 patients with biochemical evidence of progressing disease, 90% were certified as having died of prostate cancer.

Of the remaining 184 patients, 34 died in less than 6 months and so could not satisfy the criteria for 'rising markers'. Eighty-seven (47%) were certified as dying from prostatic cancer. Most of these had evidence of advanced, but stable, disease before death, but 10 patients had no evidence of skeletal metastases, and normal blood urea, acid and alkaline phosphatase at the pre-death visit.

### Discussion

Each of the methods used to measure and express disease specific mortality in prostate cancer considered here has some value and each has different disadvantages. These will be discussed in turn.

The value of the relative survival rate depends on the accuracy of the expected survival data, and on how closely the population studied matches that used to compute the expected survival rate. The age distribution of patients with prostate cancer is very different from the age distribution of the general population. Expected survival figures based on mortality in the general population must therefore be contrasted with care to observed survival in patients with prostate cancer. Here we have used 'exact age matching' to derive expected survival figures. In terms of age at presentation our series closely reflects the rates of registration in the local community, and the population studied is relatively static, most patients having lived all their lives locally. We believe that relative survival rates computed from these figures give the most objective, if indirect, measure of prostate cancer related mortality in such a group.

The data from which the figures are derived are readily accessible and the method used can be applied to any group of patients, but is clearly most applicable when considering mortality from diseases which occur, in part, in elderly patients. The main advantage of using relative survival to express mortality in prostate cancer lies in its objectivity, since no decision has to be made about each individual cause of death. The main disadvantage is that when the study population is made up of individuals from very different communities (for instance in a tertiary referral centre) the available expected survival data may be inappropriate.

As may be expected, the certified cause of death is, at best, a crude measure of disease specific mortality and it would seem that most authors are correct in avoiding the use of death certification when reporting survival. There were a number of major discrepancies identified in this study. In 10 patients (4% of those dying) clinic data suggested neither advanced nor progressive disease, and yet prostate cancer was given as the cause of death on the death certificate, while in 37% of those dying, prostate cancer was not mentioned on any part of the death certificate. In this series the death certificate was most often completed by the patient's general practitioner (who is regularly informed of his patient's progress from the prostate clinic) and so the degree of consistency in death certification data might be expected to be even lower in regions not having a dedicated prostate cancer follow-up clinic.

Despite these inconsistencies, the 'cancer specific' probability of survival at 5 years calculated from the certification data is 51.1% (Figure 5), closely matching the relative survival rate at 5 years of 50.5%. Published national mortality rates (based on certification data) have in recent years remained close to 50% of the rate of new registrations of prostate cancer (Wilson, 1987), and it is interesting to note that this observation is reflected in the relative survival rate in this series. This suggests that the certification data, although inaccurate on a relatively small scale, may begin to approximate to the genuine situation on a national scale.

Since 'referral to the hospital records' is often used to determine cause of death we were interested to see how easily a decision could be made on the basis of our own close observation of patients before death. It proved very difficult to define a set of conditions that could imply a 'cancer death'. While all urologists have witnessed patients in the terminal stages of advanced prostatic cancer and may have been well able to conclude that their patient was dying of his prostatic malignancy, the hospital record is only rarely so unequivocal.

In the absence of documented concurrent serious disease, the rising biochemical markers described here suggest a *probable* cancer specific death, but no more, and we would conclude that apart from the occasional extreme example it is not possible to identify a 'cancer death' on the basis of retrospective records.

George (1988) referred to hospital records in order to identify deaths from 'verified conditions other than prostate cancer' and these were identified as non-cancer deaths in his series. In reviewing causes of death in our series, it has proved very difficult to identify causes that exclude prostate cancer from contributing to the cause of death. In our series there were 17 deaths 'due to' other malignant neoplasms, 24 to acute myocardial infarction, one to complication of a strangulated inguinal hernia, one to rupture of an abdominal aortic aneurysm and one to alcohol induced hepatic cirrhosis. Some of these lethal processes may have been entirely independent of prostate cancer but others may not. To give one instance, three of these certified of dying of cerebrovascular or cardiovascular catastrophe had been previously treated with stilboestrol. It would be wrong not to accept that prostatic cancer may have contributed significantly to such deaths.

The possible criteria used to attribute causes to deaths on the basis of hospital records are complex and thus difficult to define and reproduce. Furthermore, if the criteria used cannot be clearly defined, interpretation of the hospital record is open to subjective bias and cannot therefore be recommended for use in the calculation of disease specific mortality.

In conclusion, the interpretation of reports of survival in prostate cancer would be facilitated by the reporting of the expected survival (calculated in a clearly defined manner on the best available population data) of each group considered. This single statistic takes into account the age of each individual in the group and best describes the age related general mortality in the group. It can be compared directly between reports from different centres, allowing the reader to judge how much any difference in observed mortality is due to prostate cancer and its treatment, and how much is due to difference in general mortality. Relative survival at a given time can be easily computed and used as an indirect but objective expression of cancer specific mortality.

The reporting of cancer specific mortality based on using retrospective records to identify 'cancer deaths' and 'deaths due to other causes' is of limited value, since the criteria used cannot be simply defined in a reproducible manner. Death certification data are confirmed to be inaccurate in the context of prostate cancer mortality studies and should continue to be avoided. Reports of mortality and survival which fail to give any information on patient age cannot be interpreted sensibly.

We gratefully acknowledge the valuable assistance of the staff of New Register House, Edinburgh, and thank the Registrar General for Scotland for permission to publish data derived from the death registry. We are grateful to Mr J.E. Newsam and Mr T.B. Hargreave for their consent to report on patients in their care and to Dr R.A. Elton of the Medical Statistics Unit of the University of Edinburgh for advice.

### References

ANNUAL REPORTS OF THE REGISTRAR GENERAL FOR SCOTLAND (1978-87). Numbers 123-133. HMSO: London.

ARMITAGE, P. & BERRY, G. (1987). Survival analysis. In *Statistical Methods in Medical Research*, 2nd edn., p. 241. Blackwell: Oxford.

GEORGE, N.J.R. (1988). Natural history of localised prostatic cancer managed by conservative therapy alone. *Lancet*, **i**, 494.

GOODMAN, C.M., BUSUTTIL, A. & CHISHOLM, G.D. (1988). Age, and size and grade of tumour predict prognosis in incidentally diagnosed carcinoma of the prostate. *Br. J. Urol.*, **62**, 576.

JOHANSSON, J., ADAMI, H., ANDERSSON, S., BERGSTROM, R., KRUSEMO, U.B. & KRAAZ, W. (1989). Natural history of localised prostatic cancer. A population based study in 223 untreated patients. *Lancet*, **i**, 799.

MERRICK, M.V., DING, C.L., CHISHOLM, G.D & ELTON, R.A. (1985). Prognostic significance of alkaline and acid phosphatase and skeletal scintigraphy in carcinoma of the prostate. *Br. J. Urol.*, **57**, 715.

PARKER, M.C., COOK, A., RIDDLE, P.R., FRYATT, I., O'SULLIVAN, J. & SHEARER, R.J. (1985). Is delayed treatment justified in carcinoma of the prostate? *Br. J. Urol.*, **57**, 724.

PAULSON, D.F. & WALTHER, P.J. (1989). Is grade or stage of primary importance in determining the outcome after radical prostatectomy for disease clinically confined to the prostate? *Br. J. Urol.*, **63**, 301.

PETO, R., PIKE, M.C., ARMITAGE, P. & 7 others (1977). Design and analysis of randomised clinical trials which require prolonged observation of each patient. II. Analysis and examples. *Br. J. Cancer*, **35**, 1

WILSON, J.M.G. (1987). Epidemiology of adenocarcinoma of the prostate. In *Adenocarcinoma of the Prostate*, Bruce, A.W. & Trachtenberg, J. (eds) p. 2. Springer-Verlag: Berlin.

WILSON, J.M.G., KEMP, I.W. & STEIN, G.J. (1984). Cancer of the prostate. Do younger men have a poorer survival rate? *Br. J. Urol.*, **56**, 391.